# Supplementary material for: Seminoma and Embryonal Carcinoma Footprints Identified by Analysis of Integrated Genome-Wide Epigenetic and Expression Profiles of Germ Cell Cancer Cell Lines
Source: PLoS One. 2014 Jun 2;9(6):e98330. doi: 10.1371/journal.pone.0098330 (PMC4041891; doi:10.1371/journal.pone.0098330)
Supplement: File S1 — ZIP file containing DMRforPairs output for significant regions. Please start from the html files. (ZIP) [file pone.0098330.s008.zip › figures/25442.pdf]

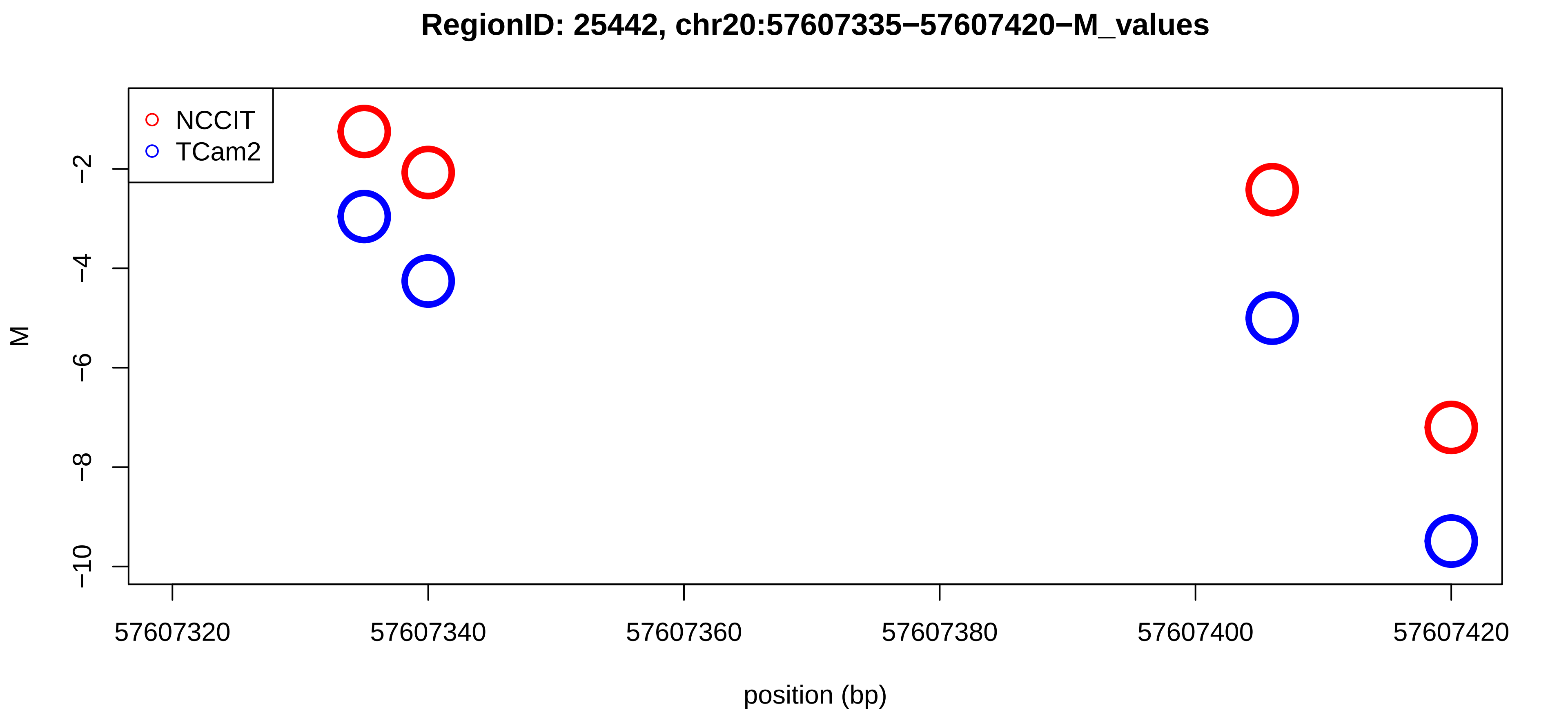
RegionID: 25442, chr20:57607335−57607420−M_values
NCCIT
TCam2
M
−2
−4
−6
−8
−10
57607320
57607340
57607360
57607380
57607400
57607420
position (bp)

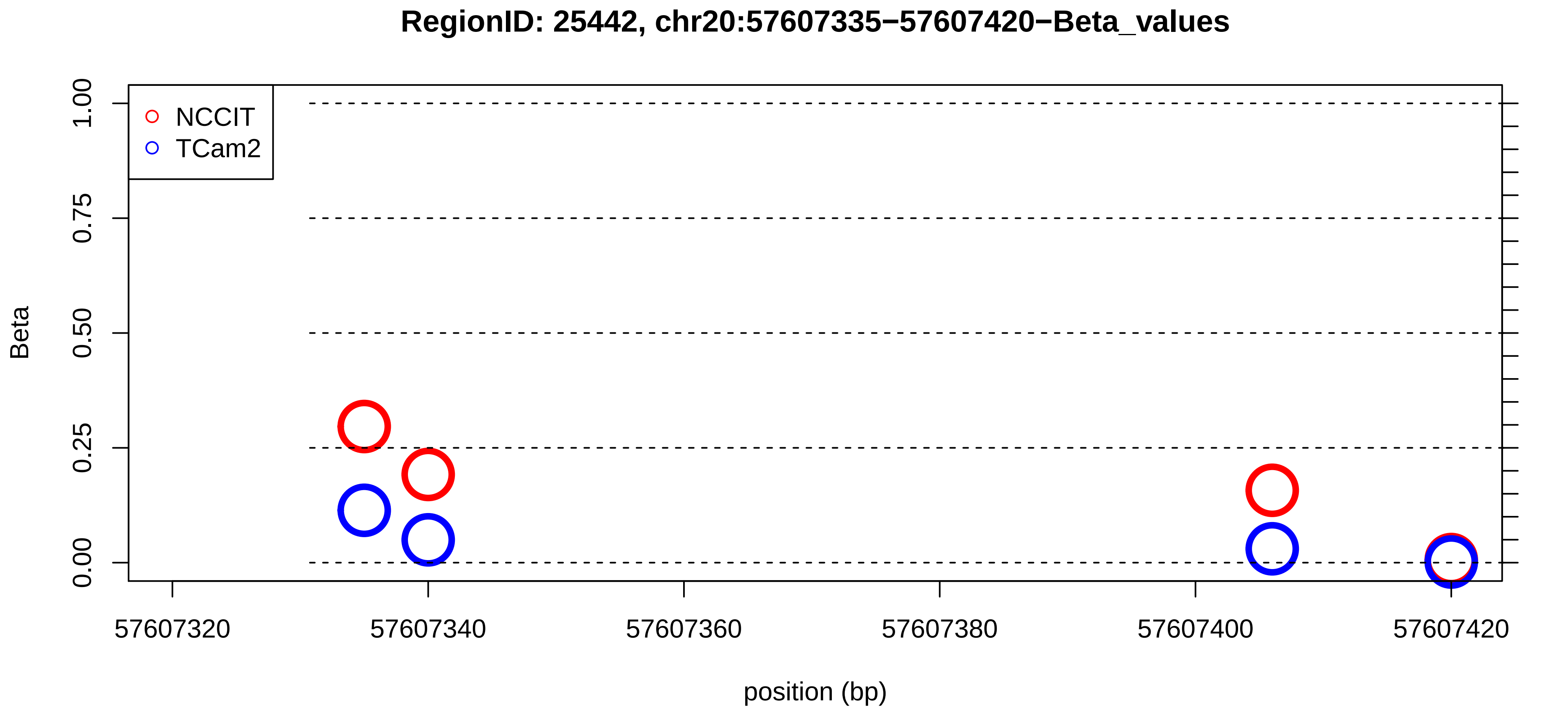
RegionID: 25442, chr20:57607335−57607420−Beta_values
NCCIT
TCam2
Beta
0.00
0.25
0.50
0.75
1.00
57607320
57607340
57607360
57607380
57607400
57607420
position (bp)
